# Oxygen Supply System Management in an Overweight Adult after 12 Months in Antarctica—Study Case

**DOI:** 10.3390/ijerph18084077

**Published:** 2021-04-13

**Authors:** Maria Radziejowska, Yevgen Moiseyenko, Paweł Radziejowski, Michał Zych

**Affiliations:** 1 Department of Innovations and Safety Management Systems, Faculty of Management, Czestochowa University of Technology, 42-200 Czestochowa, Poland; pawel.radziejowski@pcz.pl; 2 Department of Hypoxic States Investigation, Bogomoletz Institute of Physiology of National Academy of Sciences of Ukraine, 01024 Kyiv, Ukraine; moiseyenkoev@gmail.com; 3 Faculty of Health Sciences, Jan Dlugosz University, 42-200 Czestochowa, Poland; m.zych@ajd.czest.pl

**Keywords:** functional respiratory system, hypoxic state, rate of oxygen delivery

## Abstract

The aim of the study was to try to determine the functional state of the respiratory system, i.e., selected parameters and indicators of physiological systems responsible for the supply of oxygen at all stages of its delivery in people as their body weight increases from normal weight to overweight. The studies include an analysis of test results of functional respiratory system state (FSD) indicators of a 30-year-old and 170-cm tall man. Measurements of FSD were conducted two times: the first time before an expedition to Antarctica at 70 kg (normal body weight); the next measurements were taken a year later, after coming back from the expedition, at 82 kg (overweight). When analyzing the functional respiratory system state in terms of the effect of overweight it was found that the maintenance of the oxygen homeostasis in those conditions occurred at the level of a compensated hypoxic state. That is why the decision to engage in physical activity can be made only if we are sure that significant destructive additive effects of both types of hypoxic influences (from excessive body weight and from the physical activity) are not overlapping.

## 1. Introduction

Data from the World Health Organization (WHO) indicate that since 1980 the number of overweight people in the world has increased over two times [1,2]. Almost all WHO documents concerning the prevention of non-infectious diseases stress that unhealthy diet and lack of physical activity are the main risk factors of such diseases, including cardiovascular diseases, cancer, and diabetes [3].

The overall aim of WHO’s strategy to control overweight and obesity is improving and protecting health through healthy diet and physical activity [3,4,5,6]. However, effective use of physical activity as a mean of dealing with excess weight is possible only when we take into consideration all of the consequences of its influence on the human body. Many science and popular science publications considering different areas of health protection describe benefits and risks connected to physical activity [6]. It is known that safe employment of any corrective measure or therapy is possible only when we know exactly which pathophysiological mechanisms we are influencing and what compensating mechanisms are stimulated. An important aspect determining usage of physical activity is the researcher’s knowledge on the status of physiological systems responsible for supplying the working cells (including muscle cells) with oxygen, an essential factor for physical activity. A systemic approach to the analysis of respiration as a whole of all the functions of all the physiological systems responsible for supplying oxygen to cells and conditions of oxygen consumption in this structural unit of an organism allowed Professor A.Z. Kolczynska to propose the concept of a functional respiratory system [7] with its integral part—regulation of an organism’s oxygen regimes [7].

Functional respiratory system (FSD), through applying several regulatory rules, performs its basic functions: assuring appropriate levels of oxygen supply and its consumption rate, evacuating produced carbon dioxide, and securing the changing organism’s demand for oxygen. According to the FSD concept, the process of transferring the breathing gases from air to mitochondria is the subject of regulation and this is controlled by the central and autonomic nervous system and the endocrine system [8].

Undoubtedly, lower physical activity contributes to the increase in body weight [9]. However, hypodynamia is also a factor limiting a full organism’s function in the future because as the degree of overweight increases physical activity starts to be connected with significant risks of injuries and strain to the cardiovascular system.

The “hypodynamia–overweight” vicious cycle occurring as a result of being overweight requires consideration of limitations that affect oxygen supply to the overweight organism under physical effort conditions. A question arises about the effectiveness and efficiency of operation of physiological systems responsible for supplying oxygen to cells of an overweight person which are forced to work under a higher strain in the case of a larger body weight compared to the normal one.

The aim of the study was to try to determine the functional state of the respiratory system, i.e., selected parameters and indicators of physiological systems responsible for the supply of oxygen at all stages of its delivery in people as their body weight increases from normal weight to overweight and possibly obesity.

## 2. Materials and Methods

The studies include an analysis of test results of functional respiratory system state (FSD) indicators of a 30-year-old and 170-cm tall man. The tests for hypoxia were conducted in Ukraine—in the Department of Hypoxic States Investigation of the Bogomoletz Institute of Physiology, of the National Academy of Science of Ukraine. Measurements of FSD were conducted 2 times: the first time before an expedition to Antarctica at 70 kg (normal body weight, BMI = 24.22 kg/m^2^); the next measurements were taken a year later, after coming back from the expedition, at 82 kg (overweight, BMI = 28.37 kg/m^2^). The examined person worked at the Akademik Vernadsky station in Antarctica as a meteorologist. Apart from him, the team at the boarding station included 11 men. All members of the expedition had the same life style. Of the 12 members of the expedition that year, only he gained so much weight that he moved from the normal weight category to the overweight category. The remaining 11 people gained less weight (3–6 kg), and only 6 participants moved from the normal weight category to the underweight category. Body weight was measured wearing only underwear. The same calibrated scales were used before and after returning from an expedition to Antarctica (TANITA MC-780 S MA, Tokyo, Japan), which gave us the ability to measure the percentage of body fat (%). The tests were conducted according to the Declaration of Helsinki (2008) (59th World Medical Association General Assembly, Seoul, Republic of Korea, October 2008). Research on the effect of weight gain was carried out with the participation of many station crews and all the studies were complex (the functions of all systems were tested before and after the expedition) and throughout the stay, the cycles were measured quarterly, and anthropometric indicators were recorded monthly (weight, height, and percentage body fat), vital capacity, electrocardiography, clinical blood tests, daily body temperature, blood pressure, heart rate, psychological examination, as well as daily monitoring of autonomic regulation indicators and sleep quality test. The results indicated one-way changes in body weight and the examination of functions with a clear tendency towards the development of maladaptive disorders and symptoms of latent hypoxia [10,11,12,13,14]. Therefore, in order to thoroughly understand latent hypoxia and the mechanisms of association with increased overweigh, targeted studies were conducted involving the average (based on screening results) nominate with the highest weight gain.

The following indicators of external respiration and gas exchange were measured:

- tidal volume (VT), respiratory rate (f), respiratory minute volume (VE), and alveolar minute volume (VA) were investigated using Spiro unit (BTL-08 Spiro Pro, Newcastle, UK). All of the lung ventilation volumes were adjusted to the standard BTPS (body temperature, pressure, water vapor saturated) conditions.
- exhaled air gas composition (F_E_О_2_, F_A_О_2_) was investigated using Masspektrometr MX6202 (Kyiv, Ukraine).

Systolic and diastolic blood pressure (mmHg) was measured from the right arm after 15 min of rest with the patient sitting upright, using an automatic oscillometric device (Omron 705CP, Kyoto, Japan) or, if this failed, a mercury sphygmomanometer. Two measurements were taken 2 min apart from each other—if one failed a third was taken. The average of two measurements was used.

Arterial blood oxygen saturation (S_a_O_2_) and heart rate (HR) was measured using the pulse oxymeter “Oxyshuttle” (Sacramento, CA, USA). The concentration of hemoglobin in arterialized blood was determined using Auto Hematology Analyzer—КЕ-7600S (Delhi, India).

A comprehensive method was employed in the study to assess the functional state and oxygen efficiency based on the simultaneous recording of variations in the parameters of respiration, circulation, respiratory blood function, and gas exchange, followed by the analysis of partial pressure, flow rates, and oxygen supply rates at all stages of its delivery in the organism: the rate of oxygen uptake in lungs (q_і_О_2_) and alveoli (q_А_О_2_), oxygen delivery in arterial blood (q_а_О_2_) and mixed venous blood (q_ṽ_О_2_), oxygen uptake in tissues (VО_2_), oxygen partial pressure in inhaled and alveolar air (p_i_O_2_ and p_A_O_2_), and oxygen tension in arterial (p_a_O_2_) and mixed venous blood (p_ṽ_О_2_). Subjected to analysis were the parameters of the effectiveness and efficiency of organism’s oxygen regimes, quality of their regulation, ventilation (EqO_2_ = VE/VO_2_) and hemodynamic (CO/VO_2_) equivalents, and the oxygen effect of a single respiratory cycle (VO_2_/f) and systole (VO_2_/HR).

Indicators characterizing oxygen supply rate at its all stages were calculated according to the following formulas:

$$q_{I}O_{2}=F_{I}O_{2}\times \mathrm{VE},$$

where F_I_O_2_—percentage of oxygen in inhaled air; VE—respiratory minute volume;

$$q_{A}O_{2}=F_{A}O_{2}\times \mathrm{VA},$$

where F_A_O_2_—percentage of oxygen in the alveolar air; VA—alveolar minute volume;

$$q_{a}O_{2}=C_{a}O_{2}\times \mathrm{CO},$$

where C_a_O_2_—concentration of oxygen in arterial blood, CO—heart minute volume;

$$C_{a}O_{2}=1.36\cdot \mathrm{Hb}\times S_{a}O_{2},$$

where 1.36—oxygen binding capacity [15], Hb—blood hemoglobin concentration, g/L; and S_a_O_2_—arterial blood oxygen saturation, %.

Rate of blood oxygen-carrying capacity (QO_2_) was determinate according to the following formula: ${\mathrm{QO}}_{2}=1.36\times \mathrm{Hb}$.

Heart stroke volume (SV) was determined according to the Isaak Starr [16] formula for adults: SV=93+0.62×(SP−DP)−0.45×DP−0.61×age, where SP—systolic pressure (mm Hg) and DP—diastolic pressure (mm Hg), age in years. Cardiac output (CO) was determined according the formula:

CO=HR×SV,

where HR—heart rate per minute (bpm) and SV—stroke volume (mL).

$$q_{ṽ}O_{2}=q_{a}O_{2}-{\mathrm{VO}}_{2},$$

where VO_2_—oxygen uptake;

$$O_{2}=\left(F_{I}O_{2}-F_{E}O_{2}\right)\times \mathrm{VE},\;$$

where F_I_O_2_—percentage of oxygen in inhaled air; F_E_O_2_—percentage of oxygen in exhaled air; and VE—respiratory minute volume.

The analysis of oxygen supply cascade in the organism and oxygen partial pressure was realized thanks to using a mathematical model of modes of the oxygen regulatory system [8]. This enabled the determination of qualitative and quantitative characteristics of oxygen homeostasis at normal body weight and overweight. For calculations of the parameters of oxygen supply on every stage of its delivery, ventilation volumes were adjusted to the STPD conditions (the gas volume has been expressed as if it were at standard temperature (0 °C), standard pressure (760 mmHg absolute), and dry). Oxygen partial pressures in inhaled and alveolar air were determined according to the following formulas:

$$p_{I}O_{2}\;=\;\frac{\left(B-b\right)\times F_{I}O_{2}}{100},$$

where B—atmospheric pressure and b—steam pressure;

$$p_{A}O_{2}\;=\;\frac{\left(B-47\right)\times F_{A}O_{2}}{100},$$

To determine oxygen tension in arterial and mixed venous blood (p_a_O_2_ and p_ṽ_O_2_, respectively), a nomogram of oxyhemoglobin dissociation curve at known levels of arterial blood (S_a_O_2_) and mixed venous blood (S_ṽ_O_2_) saturation were used. While the level of mixed venous blood saturation (S_ṽ_O_2_) was determined according to the following formula:

$$S_{ṽ}O_{2}\;=\;\frac{C_{ṽ}O_{2}}{1.36\times \mathrm{Hb}},\;$$

where C_ṽ_O_2_—concentration of oxygen in mixed venous blood, calculated according to the following formula:

$$C_{ṽ}O_{2}\;=\;\frac{q_{ṽ}O_{2}}{\mathrm{CO}}$$

For the purpose of theoretical studies on the influence of excessive body weight on the effectiveness of oxygen consumption in working tissues, computer models of functional respiratory system were used [8].

## 3. Results

Being overweight and obese results from improper or excessive accumulation of fat tissue that can have harmful effect on health [17]. The Body Mass Index (BMI)—a result of dividing body mass in kilograms by square height in meters is often used to determine overweight and obesity level in adults [5]. Assuming that normal body weight of a person 170-cm tall is 70 kg, 80 kg meaning overweight, 90 kg—first degree obesity, 105 kg—second degree obesity, and 120 kg—severe obesity, it is possible, using computer model calculations of functional respiratory system, to determine how the oxygen consumption rate per 1 kg of body mass would decrease when the remaining FSD parameters will stay the same (Table 1).

The studies showed that the value of the parameter characterizing intensity of oxygen uptake in an adult 170-cm tall person at normal body weight equals 1 MET (metabolic equivalent), i.e., according to the requirements of meeting the energy demand per 1 kg of body mass, functional respiratory system supplies 3.48 mL of oxygen per minute to organism’s cells. MET—metabolic equivalent; 1 MET means utilization of O_2_ at rest and equals 3.5 mL O_2_/kg body mass/min or 1 kcal/kg/h or 4.184 kJ/kg/h [18]. As shown by the parameters in Table 1 with an increase in body weight and no changes in pulmonary ventilation, gas exchange in lungs, and functional parameters of circulatory system and hematopoiesis, when body weight reaches 120 kg (severe obesity) the oxygen consumption per 1 kg of body mass decreases almost twice, what can indicate terminal degree of hypoxia [8,19,20,21,22,23].

Based on the analysis, under the conditions of overweight or obesity, when the organism is at risk of possible secondary tissue hypoxia, it can also be assumed that mechanisms compensating the hypoxia have to be activated. However, do the compensatory changes really occur under such conditions? To answer this question, we conducted the analysis of laboratory tests results of a person who gained 12 kg over a year. The percentage of body fat increased from 24.83% to 34.91%.

Comparing test results at normal body weight (70 kg, BMI = 24.22 kg/m^2^) and overweight (82 kg, BMI = 28.37 kg/m^2^), if under the conditions of overweight activation of compensatory mechanisms will be observed, should allow to determine the reasons for the decrease in oxygen consumption per 1 kg of body weight found at overweight using the computer models or to correct this hypothesis.

Already preliminary overview of test protocols led to an observation of lowered parameters of pulmonary ventilation registered under the conditions of ATPS (the gas volume has been expressed as if it were saturated with water vapor at the ambient temperature and barometric pressure). Parameters of gas exchange did not significantly differ but parameters of circulation at overweight were worse (HR higher by almost 21 bpm, stroke output lower by 10 mL in comparison to results at normal body weight) (Table 2). Conditions enabling to maintain optimal oxygen homoeostasis at overweight were worse as indicated by significant hypoxemia—decrease in p_a_O_2_ from 93 to 83.8 mm Hg (see Table 2). The decrease in the level of working capacity can be indirectly judged by the existing dysfunction of the mitochondrial apparatus, which was found in all members of the expedition in addition to the examined one [13]. This is confirmed by the data of bicycle ergometric testing carried out before and after being a member of the expedition in Antarctica [10].

Using the FSD model it was possible to calculate selected parameters of oxygen homoeostasis at normal body weight and overweight in the same person (Table 3).

**Figure 1:**
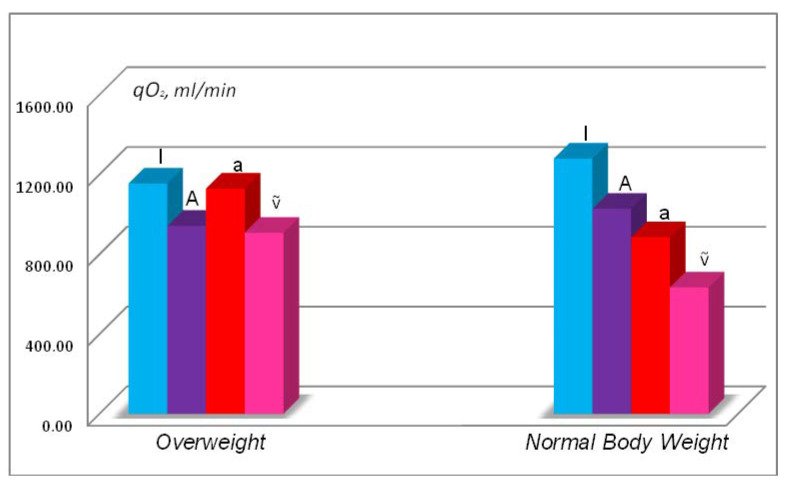
Rate of oxygen uptake in lungs (q_I_O_2_), oxygen uptake in alveoli (q_A_O_2_), oxygen delivery in arterial blood (q_a_O_2_), and oxygen delivery in mixed venous blood (q_ṽ_O_2_) in the same 170-cm tall at overweight (82 kg) and at normal body weight (70 kg).

FSD analysis of the subject from the angle of the influence of overweight enabled to conclude that maintaining oxygen homoeostasis under these conditions is at the level of compensated hypoxic condition according to the classification of hypoxic conditions [8,21,22,23]. This is indicated by lowered parameters of pulmonary ventilation and alveolar ventilation in comparison to the baseline (see Table 3) and compensation of hypoxic condition reached through increased function of circulatory system and hematopoiesis thanks to increased minute heart volume and blood oxygen capacity. This FSD actions led to relative balance of the hypoxic condition when the level of total oxygen uptake lowered by only 12%. In certain instances, changes on such level can be regarded as measuring errors if not changes of the supply rate cascade due to being overweight (Figure 1).

Figure 1 shows that in an overweight person the oxygen supply rate cascade has an abnormal (distorted) form—rate of oxygen uptake in alveoli (q_A_O_2_) is lower than the rate of oxygen delivery in arterial blood (q_a_O_2_). Such a phenomenon of compensation of the hypoxic condition caused by intensification of circulatory system function and hematopoiesis can indicate latent (hidden) or compensated hypoxic condition [8,19,20,21,22,23].

The oxygen supply rate cascade (expressed per 1 kg of body mass) were also abnormal. Rate of oxygen uptake by tissues when overweight significantly drops (by 25% in comparison to normal body weight, change from 3.58 mL/min/kg to 2.70 mL/min/kg), i.e., it equals 0.75 MET. Such a change of this parameter gives a lot to think about. Even intensification of compensatory mechanisms of the circulatory system at overweight is not enough to maintain oxygen uptake per 1 kg at the same level as in the case of normal body weight (see Table 4).

Parameters of the efficiency of pulmonary ventilation differed only slightly—ventilation equivalent (VE/VO_2_) increased by only 2.5% (change from 28.69 to 29.41) and oxygen effect of one respiratory cycle decreased by 5.4% (change from 15.68 to 14.83). The high increase in the intensity of compensatory mechanisms of the circulatory system is also indicated by changing parameters of FSD efficiency. Hematopoiesis equivalent (CO/VO_2_) at overweight was 16% higher (change from 19.66 to 22.85) and the oxygen effect of heart contraction (VO_2_/HR) was 28% lower (change from 3.18 to 2.30) in comparison to normal body weight. This means that at overweight, organs of the circulatory system had to work with higher intensity, requiring higher increase in the stroke volume due to increased heart rate in order to reach the level of oxygen uptake lower by 25% in comparison to the norm what indicates that being overweight is a hazard to normal function of the cardiovascular system.

## 4. Discussion

Antarctica is a unique place to study health conditions under the influence of environmental factors on the organism in pure form [10,11,12,13,14,24].

Medical research in Antarctica opens up new horizons of understanding across a spectrum of individual health conditions under stress and unusual environmental conditions [24]. The series of the retrospective and prospective studies demonstrated the strong influence of extreme environmental factors and stress in the absence of man-made factors on health parameters, with clearly defined individually patterned reactions [10,11,12,13,14,24,25,26].

The 12-month stay in Antarctica was used as a long-term impact of a specific test load, because the complex factors affecting these conditions are unique and avoids technogenic impact.This allowed us to determine the level of oxygen delivery in overweight people, which was the result of the impact of stressors. The 12-month stay in Antarctica was used as a long-term impact of a specific test load, as the complex of influencing factors in these conditions allowed to neutralize the biorhythmological fluctuations in body weight during the year. This allowed us to determine the level of oxygen delivery in an overweight person, which was the result of the influence of a unique complex of stressors found in Antarctica. In Europe, it is not possible to model conditions that are devoid of technogenic influences as is possible with Antarctic conditions. Also, factors provoking the development of disorders of oxygen delivery at all stages of mass-transfer may be due to disorders of the central regulation of desynchronizes as impaired sleep–wakefulness rhythm disorders and autonomic regulation [10,11,12,13,14]. All of the points above, including the conditions of forced hypodynamia, contribute to body weight gain and the greater the weight gain, the more pronounced the disturbances in the level of oxygen delivery due to an increase in the load on the heart and the function of external respiration.

Since the time domains of the physiological hypoxic ventilatory response (HVR) were identified, considerable research effort has gone toward elucidating the underlying molecular mechanisms that mediate these varied responses [7,8,19,27].

Calculating the functional respiratory system’s parameters for the same person at normal body weight and overweight, based on the results of laboratory tests, allowed for the determination of some mechanisms compensating the hypoxic conditions that occurred when this person became overweight after the 12-month stay in Antarctica. It was noted that when the person was overweight oxygen uptake per 1 kg of body mass decreased to the level of 0.75 MET and it was connected to a significant increase of hemoglobin levels (see Table 1). However, data reported by Loftin M. et al. [28] on the level of oxygen uptake in normal-weight and overweight persons indicated that values of this parameter (VO_2_, L/min^−1^) were similar in the severely overweight and normal-weight female youth). Although, during physical activity O_2_ shortage was significantly higher in severely overweight persons (0.75 ± 0.15 L) in comparison to the group of normal-weight persons (0.34 ± 0.13 L). Studies of Panwar B. et al. [29] assessing the risk of stroke depending on hemoglobin concentration showed that as the BMI (thus, the degree of overweight) increases the blood hemoglobin concentration tends to decrease. However, in our studies the hemoglobin concertation, hence blood oxygen capacity, increased and can be considered a specific reaction to low level hypoxia that usually stimulates erythropoiesis.

In modern literature concerning oxygen uptake in overweight persons, determination of oxygen uptake efficiency slope (OUES), a new index of cardiorespiratory functional reserve derived from the relation between oxygen uptake and minute ventilation during incremental exercise, prevails. Oxygen uptake efficiency slope (OUES), as a submaximal measure of cardiorespiratory functional reserve, is derived from the relation between oxygen uptake (Vo_2_, mL/min) and minute ventilation (Ve, liters/min) during incremental exercise and is determined by Vo_2_ = a log Ve + b, where a = OUES, which shows the effectiveness of Vo_2_ [30]. Determination of the efficiency of oxygen uptake in overweight persons is already a golden standard of modern physiology [31,32,33,34,35,36,37]. It is an indicator determining an organism’s oxygen consumption under dynamic conditions of physical activity. Drinkard B. et al. [33] found that OUES adjusted for lean body mass was shown to be lower in overweight adolescents. In addition, the wide interindividual variation, the magnitude bias, and the intensity dependence of the OUES impede its clinical utility for assessing the fitness level of severely overweight adolescents. In our studies conducted under static conditions (at rest and at normal weight and overweight), to determine the effectiveness of lung ventilation, the ventilation equivalent (EqO_2_ = VE/VO_2_) was used. Calculations carried out for the same person at normal body weight and overweight showed that changes of the VE/VO_2_ ratio or the oxygen effect of one respiratory cycle did not have a considerable significance. Whereas the indicators describing the economy of circulatory system’s function were more deviated in the case of overweight in comparison to those of normal weight. The hematopoiesis equivalent (CO/VO_2_) significantly increased and this means that in an overweight person consumption of 1 L of oxygen requires that the heart pumps 16% more arterial blood in comparison to a normal-weight person.

Blood pressure at overweight increased to pre-pathological values [38] resulting in lowering of SV. However, in the context of higher significance of heart rate, the minute volume (CO) increased by 310 mL. The increased CO and higher blood oxygen capacity at overweight resulted in an abnormal form of the oxygen supply to tissues rate cascade (rate of oxygen delivery in arterial blood was higher than the rate of oxygen uptake in alveoli). Such form of the oxygen supply cascade is usually observed in the mountains, under the conditions of lowered oxygen partial pressure in the inhaled air [21,22]. Along with significant decrease in oxygen consumption at rest per 1 kg of body mass when the subject was overweight it can suggest the influence of the long-term chronic intermittent hypoxia. Siques P. et al. [39] indicated that overweight and obesity impair the activity of adaptive mechanisms during the process of acclimatization in the mountains. According to San Martin R. et al. [40] obesity, or being overweight, plays a major role in the development of the high-altitude illnesses (acute mountain sickness, hypoxic pulmonary hypertension, and chronic mountain sickness). This association could be rooted in the interactions between obesity-related metabolic alterations and critical ventilation impairments due to obesity, which would aggravate hypobaric hypoxia at high altitudes, leading to hypoxemia, which is a trigger for developing high-altitude diseases. Our studies also showed a significant increase in arterial blood saturation at overweight in comparison to the analogous indicator at normal body weight, that resulted in lowering of the paO_2_ by almost 10 mm Hg (see Table 2).

## 5. Conclusions

Calculations of functional state of the respiratory system parameters based on laboratory test results of the same person at normal body mass and overweight enabled the determination of some mechanisms of compensating the hypoxic condition occurring in overweight persons. The role of excess body weight in the development of hypoxic condition in the organism and compensatory role of the circulatory system were supported. The decision to engage in aerobic workouts that are so effective in reducing body weight can be made only if we are sure that significant destructive additive effects of both types of hypoxic influences (the already existing one resulting from excessive body weight and the one resulting from hypermetabolic hypoxia, i.e., strain hypoxia) are not overlapping.

## 6. Practical Recommendation

As we can see, in the case of overweight person compensation of hypoxic state is still present at rest, because the level of paO_2_ still has not reached its critical level that ranges from 50 to 65 mm Hg for different age groups [41]. However, will the symptoms of hypoxia deepen with increasing body weight? That is why the decision on starting aerobic workouts, so efficiently facilitating the process of losing weight [42], can be made only when we are sure that significant destructive additive effects of the two types of hypoxic influences (the one already existing due to excessive weight and the one resulting from hypermetabolic hypoxia—the load hypoxia) are not overlapping. An important indicator of an overweight person’s condition would be fixation of arterial blood saturation using physical activity directed to development of higher minute lung ventilation and its efficiency in supplying oxygen to the cells of the working organism. In our opinion, such a risk should be assessed individually in every instance, based on the test results including indicators of the function of functional respiratory system whose main task is to deliver oxygen from the surrounding air to the working cells, oxygen utilization, and removing carbon dioxide from the organism.

## Figures and Tables

**Table 1:** Rates of oxygen uptake and its supply at every stage of its delivery in mL/min per 1 kg of body mass (mL/min·kg^−1^).

Parameter	70 kg	80 kg	90 kg	105 kg	120 kg
Oxygen uptake	3.48	2.87	2.55	2.19	1.91
Rate of О_2_ uptake in lungs	22.84	19.99	17.77	15.23	13.32
Rate of О_2_ uptake in alveolar surface of lungs	17.13	14.99	13.32	11.42	9.99
Rate of О_2_ delivery in arterial blood	12.51	10.94	9.73	8.34	7.30
Rate of О_2_ delivery in mixed venous blood	9.23	8.07	7.18	6.15	5.38

**Table 2:** Laboratory test results of the same person at normal body weight (1) and overweight (2).

Laboratory Test Results	1	2
V_E_O_2 ATPS_, L/min	7.2	6.5
F_E_O_2_,%	16.8	16.9
F_A_O_2_,%	15.8	16
p_I_O_2_, mm Hg	159	159
p_A_O_2_, mm Hg	120	100
*f*, breaths/min	16	14.9
HR, bpm	75	96
Systolic blood pressure, mm Hg	122	129
Diastolic blood pressure, mm Hg	71	85
SV, mL	63.2	52.6
Hb, g/L	140	173
SaO_2_,%	97	92
РаО_2_, mm Hg	93	83.8
S_ṽ_O_2_,%	71.60	71.57

**Table 3:** Calculated functional indicators of the functional respiratory system state in the same person at normal body weight and overweight.

Calculated Functional Indicators	Normal Body Weight	Overweight	Difference
VE_BTPS_, mL/min	7.27	6.57	−0.71
VE_STPD_, mL/min	6.12	5.53	−0.60
VA_BTPS_, mL/min	5.85	5.36	−0.49
CO, mL/min	4740	5050	310
Blood oxygen capacity, mL/L	190.4	235.28	44.88
VT_BTPS_, mL	450.00	436.24	−13.76
VO_2_, mL/min	250.92	221	−29.92

**Table 4:** Indicators of oxygen uptake rate per 1 kg of body mass at all stages of oxygen supply in the same person at normal body weight (1) and overweight (2).

Oxygen Uptake Rate	1	2	Difference
Oxygen uptake in tissues per 1 kg of body mass, mL/min/kg	3.58	2.70	−0.89
Rate of oxygen uptake in lungs, mL/min/kg	15.60	14.08	−1.52
Rate of oxygen uptake in alveoli, mL/min/kg	12.54	11.50	−1.04
Rate of oxygen delivery in arterial blood, mL/min/kg	10.79	13.77	2.98
Rate of oxygen delivery in mixed venous blood, mL/min/kg	7.73	11.07	3.34

## Data Availability

Not applicable.
